# Disrupted endothelial cell heterogeneity and network organization impair vascular function in prediabetic obesity

**DOI:** 10.1016/j.metabol.2020.154340

**Published:** 2020-10

**Authors:** Calum Wilson, Xun Zhang, Matthew D. Lee, Margaret MacDonald, Helen R. Heathcote, Nasser M.N. Alorfi, Charlotte Buckley, Sharron Dolan, John G. McCarron

**Affiliations:** a Strathclyde Institute of Pharmacy and Biomedical Sciences, University of Strathclyde, 161 Cathedral Street, Glasgow G4 0RE, UK; b Department of Biological and Biomedical Sciences, Glasgow Caledonian University, Glasgow G4 0BA, UK

**Keywords:** 2-APB, 2-aminoethoxydiphenyl borate, 4-DAMP, 1,1-dimethyl-4-diphenylacetoxypiperidinium iodide, ACh, acetylcholine, ANOVA, analysis of variance, HFD, high-fat diet, IEL, internal elastic lamina, L-NAME, L-N^G^-Nitro arginine methyl ester, IP_3_, inositol triphosphate, MEP, myoendothelial projection, NO, nitric oxide, RuR, ruthenium red, SD, standard diet, SNP, sodium nitroprusside, Endothelial cells, Heterogeneity, Vasodilation, Networks, Obesity

## Abstract

**Background:**

Obesity is a major risk factor for diabetes and cardiovascular diseases such as hypertension, heart failure, and stroke. Impaired endothelial function occurs in the earliest stages of obesity and underlies vascular alterations that give rise to cardiovascular disease. However, the mechanisms that link weight gain to endothelial dysfunction are ill-defined. Increasing evidence suggests that endothelial cells are not a population of uniform cells but are highly heterogeneous and are organized as a communicating multicellular network that controls vascular function.

**Purpose:**

To investigate the hypothesis that disrupted endothelial heterogeneity and network-level organization contribute to impaired vascular reactivity in obesity.

**Methods and results:**

To study obesity-related vascular function without complications associated with diabetes, a state of prediabetic obesity was induced in rats. Small artery diameter recordings confirmed nitric-oxide mediated vasodilator responses were dependent on increases in endothelial calcium levels and were impaired in obese animals. Single-photon imaging revealed a linear relationship between blood vessel relaxation and population-wide calcium responses. Obesity did not alter the slope of this relationship, but impaired calcium responses in the endothelial cell network. The network comprised structural and functional components. The structural architecture, a hexagonal lattice network of connected cells, was unchanged in obesity. The functional network contained sub-populations of clustered specialized agonist-sensing cells from which signals were communicated through the network. In obesity there were fewer but larger clusters of sensory cells and communication path lengths between clusters increased. Communication between neighboring cells was unaltered in obesity. Altered network organization resulted in impaired, population-level calcium signaling and deficient endothelial control of vascular tone.

**Conclusions:**

The distribution of cells in the endothelial network is critical in determining overall vascular response. Altered cell heterogeneity and arrangement in obesity decreases endothelial function and provides a novel framework for understanding compromised endothelial function in cardiovascular disease.

## Highlights

• The endothelium is an assembly of exquisitely organized cells in a highly-refined network that is essential to function.
• In obesity, the network is disorganized into fewer, larger sensory clusters with increased path lengths between clusters
• The disorganized endothelial network resulted in deficient encoding of vasoactive stimuli to population level Ca^2+^ responses
• Changes in the network organization of endothelial cells contributes to obesity-related vascular dysfunction.

## 1 Introduction

Obesity is a major risk factor for cardiovascular diseases such as hypertension and stroke [[1], [2], [3]]. Each of these diseases is precipitated by alterations in the endothelial cell lining of blood vessels [4]. The most frequently reported effect of obesity on endothelial function is a reduction in the ability of the cell layer to control vascular tone. Clinically, this endothelial dysfunction is indicated by reductions in hyperemia-induced forearm blood flow [5,6]. Hyperemic flow is also impaired in obese children [7], even in the absence of insulin resistance [8], and short-term studies demonstrate reversible impairment of endothelium-dependent vasodilation with weight gain and loss [9,10]. Thus, irrespective of age and in the absence of co-existing diseases, obesity is associated with impaired endothelial function.

Impaired endothelium-dependent function is also observed in various animal models of obesity, including obese Zucker [11,12] and JCR:LA-cp rats [13], and rats fed a high-fat diet [[14], [15], [16]]. However, the mechanisms underlying endothelial dysfunction in obesity are unclear. Increased production of reactive oxygen species, which inactivates endothelial-derived nitric oxide (NO) [16,17], may explain reduced agonist-induced vasodilation in obese animals. Impaired endothelium-dependent vasodilation in obesity may also arise from changes in potassium channel activity [[18], [19], [20]], NO release [20], or how perivascular adipose tissue modulates endothelial function [[21], [22], [23]]. The number of diverse signaling mechanisms proposed to account for endothelial dysfunction in obesity demonstrates how complex changes occur in this cell layer, and point to changes in another regulator that is common to each dysfunction.

Intracellular Ca^2+^ is a second messenger that relays signals from numerous endothelial cell membrane receptors to effector proteins that govern vascular function [24]. For example, endothelial Ca^2+^ levels control endothelial derived hyperpolarizing factor, and regulate the synthesis and release of vasoactive mediators such as NO (reviewed [25]). Ca^2+^ signals initiate when one or a few Ca^2+^ channels open to permit an influx of the ion into the cytoplasm. The resulting elevation in cytoplasmic Ca^2+^ can remain localized around the channel(s) or can grow, by recruitment of neighboring channels, into Ca^2+^ waves that propagate within cells or between cells. Studies of human arteries highlight a deficit in subcellular and whole-cell endothelial Ca^2+^ signals in obesity [26,27], but mechanistic insight into the disruption of signaling is conflicting (subcellular) [27,28] or absent (whole-cell).

Our recent work shows that endothelial control of vascular function is driven by cellular heterogeneity and large-scale, multicellular network Ca^2+^ dynamics [29]. Even within small vascular regions, endothelial cells are diversified and arranged in a network of signal detection sites [30]. Distinct clusters of cells are tuned to detect specific activators, and different clusters detect different stimuli. This arrangement allows the endothelium to detect and process multiple stimuli in parallel and to generate stimulus-specific responses [[30], [31], [32], [33]]. These findings, together with studies that describe molecular [[34], [35], [36], [37], [38], [39], [40], [41]] and functional heterogeneity [42] in endothelial cells across vascular beds, and within single vessel segments, raise the possibility that dysfunctional vascular responses could arise from altered endothelial cell heterogeneity and disrupted network dynamics. Indeed, altered multicellular network behavior underlies disease development in a variety of physiological systems. For example, network dysfunction precedes the appearance of the earliest markers of neurodegenerative conditions [43,44], and contributes to islet failure in diabetes [[45], [46], [47]]. The role for endothelial heterogeneity in the pathophysiology of vascular disease is currently unknown.

Given the importance of endothelial function to the health problems in obesity and the absence of information on the pathophysiological correlate of endothelial cell heterogeneity, we investigated networked Ca^2+^ dynamics in large populations of endothelial cells in intact blood vessels. We show that agonist-evoked, endothelium-dependent vasodilation arises from network-level interactions that occur among a heterogeneous endothelial cell population, and that this network-level control is impaired in prediabetic obesity. Obesity alters the network, leading to a reduction in the size of the agonist-sensitive cell population, an increase in the clustering of sensory cells, and an increase in the communication distance between cell clusters. These changes in endothelial cell network dynamics impairs the collective endothelial response and provides a new framework for understanding vascular dysfunction in obesity.

## 2 Materials and methods

All data underpinning this study is available from the authors upon reasonable request. An expanded Material and Methods section can be found in the Supplemental Materials.

### 2.1 Animal models

All animal care and experimental procedures were conducted in accordance with relevant guidelines and regulations, with ethical approval of the University of Strathclyde Local Ethical Review Panel and were fully licensed by the UK Home Office regulations (Animals (Scientific Procedures) Act 1986, UK) under Personal and Project License authority. Studies are reported in compliance with the ARRIVE guidelines [48]. Twenty-eight male Wistar rats (9 weeks of age, weighing 349 ± 11 g) were split into two weight-matched groups and fed either a standard diet (SD group) or a high-fat diet (HFD group) for 24 weeks (see Supplementary Materials for full details). Only male rats were used to limit variability.

### 2.2 Experimental techniques

First-order mesenteric arteries from SD- and HFD-fed rats were studied using pressure myography, and high-resolution single photon Ca^2+^ imaging of *en face* artery preparations with and without simultaneous assessment of vascular tone. First-order mesenteric vessels were selected as a widely used model of vascular and endothelial function since there is a wealth of published background information to aid experimental design and data interpretation. For pressure myograph experiments, we used the open source pressure myograph system, VasoTracker [49] and recorded outer vessel diameter in response to various treatments at 70 mmHg and 37 °C. For Ca^2+^ imaging experiments, arteries were opened longitudinally, and endothelial cells were preferentially loaded with the Ca^2+^ indicator, Cal-520/AM (5 μM). Network-level Ca^2+^ signaling was imaged using a high (0.8) numerical aperture 16× objective (~0.8 μm^2^ field of view). This 16× objective also permitted quantification of vascular contractility in opened arteries using edge-detection algorithms [50]. Subcellular Ca^2+^ signaling was imaged using a high (1.4) numerical aperture 100× objective. Network-level and subcellular Ca^2+^ signaling was assessed using custom Python software [30,51]. Ca^2+^ responses were evoked by agonists or photolysis of caged inositol triphosphate (IP_3_). Images were acquired at either 10 Hz (agonist- or caged IP_3_-evoked activity) or 20 Hz (spontaneous activity).

### 2.3 Statistics and data analysis

Summary data are presented in text as mean ± standard error of the mean (SEM), and graphically as mean ± SEM or individual data points with the mean indicated. Graphically, paired data points are indicated by connecting lines. Concentration-response data were computed according to a three-parameter dose–response model. Data were analyzed using paired *t-*tests, independent 2-sample *t-*tests (with Welch's correction as appropriate), ordinary or repeated measures two-way ANOVA with multiple comparisons as appropriate and as indicated in the respective figure or table legend. All statistical tests were two-sided. A p value of <0.05 was considered statistically significant.

## 3 Results

### 3.1 Impaired endothelium-dependent vasodilation in obesity

**Fig. 1:**
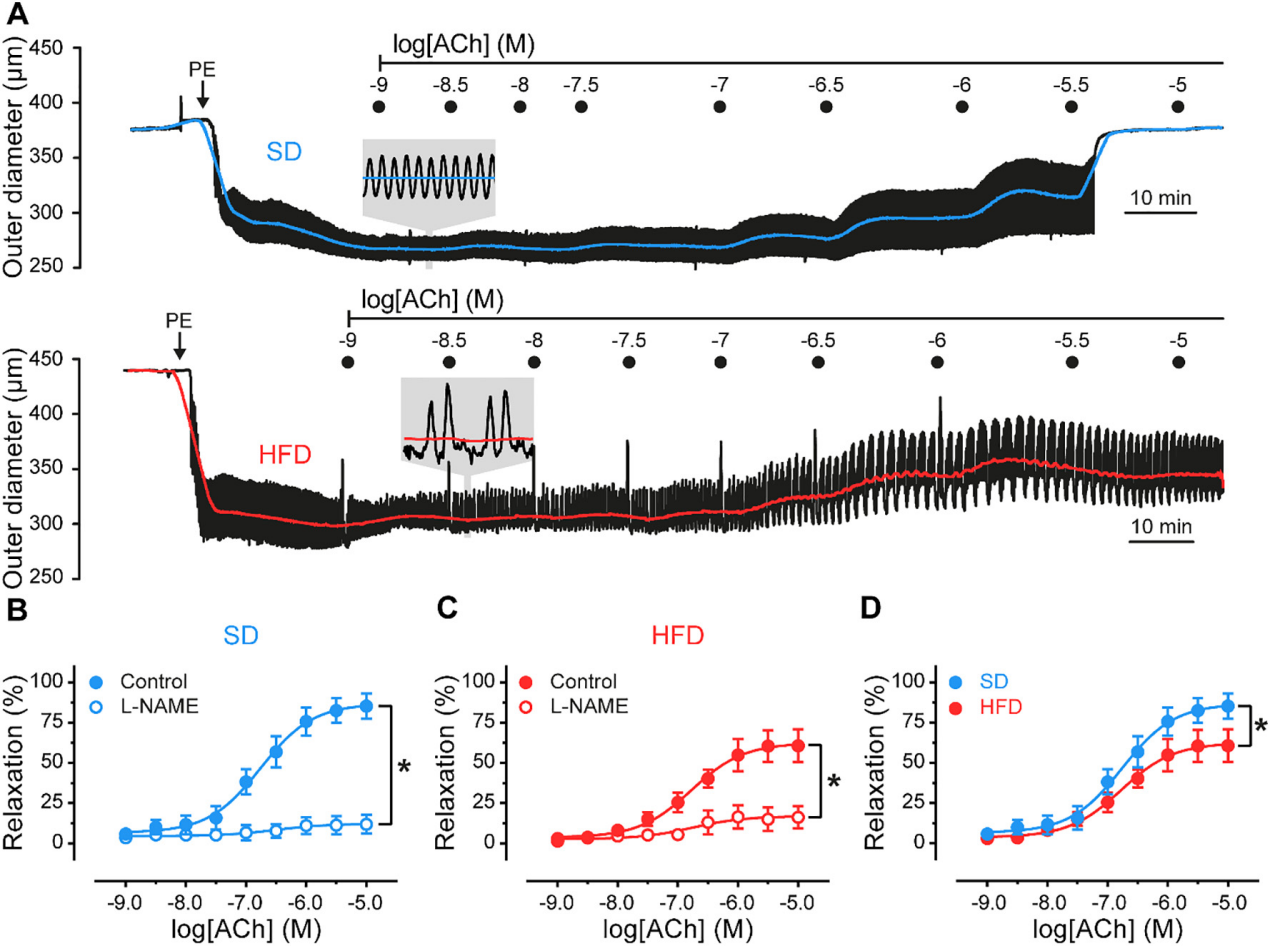
A high-fat diet impairs nitric oxide-mediated vasodilation. A) Representative mesenteric artery outer diameter traces in response to increasing concentrations of ACh. PE was continuously superfused and ACh was delivered intraluminally using a 10 cm H_2_O pressure gradient (~200 μl min^−1^). B–D) Summary of diameter data comparing the concentration-dependent responses of arteries, from rats fed a standard diet (SD, blue) and a high-fat diet (HFD, red), to intraluminal ACh in the absence (filled circles) and presence (open circles) of the nitric oxide synthase inhibitor, L-NAME (100 μM). Data are mean ± SEM (n = 7 to 9). *p < .05, by comparison of sigmoidal fit parameters (top of sigmoid) using ordinary two-way ANOVA with Tukey's post hoc test. Additional data shown in Fig. S1 and tabulated in Table S2.

Rats on a high-fat diet developed obesity, without complications associated with diabetes (Fig. S1, Table S1). To examine endothelial function in this model of obesity (see Supplementary Material for full details), we first assessed endothelium-dependent vasodilation to acetylcholine (ACh). ACh evoked concentration-dependent relaxations of established tone (PE, 300 nM–1 μM) in mesenteric arteries from SD and HFD rats (Fig. 1A). In each group (SD & HFD), ACh-induced relaxations were inhibited by the selective M_3_ muscarinic receptor antagonist, 1,1-dimethyl-4-diphenylacetoxypiperidinium iodide (4-DAMP, 1 μM), or the NO synthase inhibitor, L-N^G^-Nitro arginine methyl ester (L-NAME, 100 μM), each applied intraluminally (Figs. 1B–C, S2 and Table S2). The inhibition of ACh-evoked relaxation by L-NAME was slightly enhanced by the additional presence of the K^+^ channel blockers TRAM34 (1 μM) and apamin (100 nM; Table S2, Fig. S2) in SD and HFD groups. These results suggest that the major mechanism underlying relaxation is endothelial NO production.

The muscarinic-receptor mediated, NO-dependent relaxations were impaired in mesenteric arteries from the HFD group (Fig. 1D). However, there was no change in relaxation to the endothelium-independent NO donor, sodium nitroprusside (SNP, 10 μM; Table S2), suggesting that the processes involved in the production of NO are impaired in HFD-fed rats.

Endothelial production of NO is generally considered to be a Ca^2+^-dependent process [25]. To determine if ACh-induced, NO-mediated vasodilation required an increase in endothelial Ca^2+^, we measured endothelial Ca^2+^ levels and vasoactivity before and after block of NO production or buffering endothelial Ca^2+^. Inhibiting NO synthesis using L-NAME (100 μM) enhanced PE-evoked contractions and inhibited ACh-evoked relaxations but had no effect on endothelial Ca^2+^ levels in SD or HFD groups (Fig. S3 and Table S3). Preventing endothelial Ca^2+^ changes, by buffering Ca^2+^ with BAPTA, reduced basal intracellular Ca^2+^ levels, potentiated PE-induced contractions and inhibited ACh-induced relaxations in SD- and HFD-fed groups (Fig. S3 and Table S4). The latter results demonstrate that endothelial Ca^2+^ signaling is required for endothelium-dependent relaxations.

### 3.2 Population-level dysfunction of endothelial Ca^2+^ signaling in obesity

**Fig. 2:**
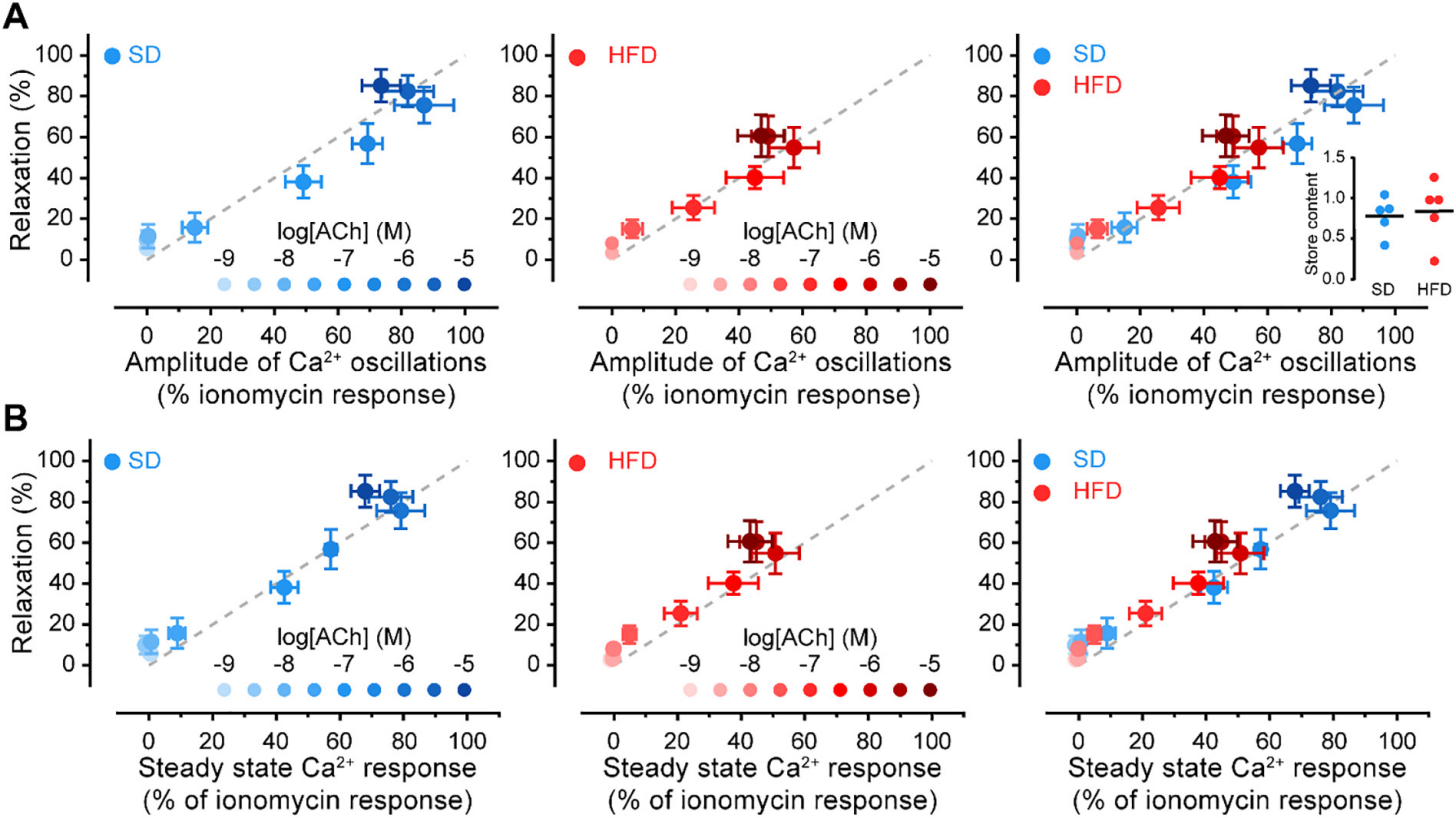
ACh-evoked endothelial Ca^2+^ levels and vascular relaxation are linearly proportional. A–B) Relationship between the amplitude of Ca^2+^ oscillations (A) or steady-state Ca^2+^ levels (B) and blood vessel relaxation from rats fed a standard diet (SD, blue) or a high-fat diet (HFD, red). The grey line represents a theoretical 1:1 correspondence between the Ca^2+^ parameter (x-axis) and relaxation. Inset in A plots the Ca^2+^ store content (area under the curve of ionomycin-evoked Ca^2+^ response) for SD and HFD groups.

To examine the precise relationship between endothelial Ca^2+^ signaling and endothelium-dependent relaxation, we examined population-wide Ca^2+^ activity using high spatiotemporal resolution, wide-field single photon imaging (50–100 cells). We used a full ACh concentration series (1 nM–10 μM) to activate the cells while Ca^2+^ activity was recorded. The Ca^2+^ responses obtained in these experiments were normalized to maximal responses induced by ionomycin (1 μM), which were statistically similar in SD and HFD (Fig. 2A, inset), and compared to the extent of relaxation at the same ACh concentration. As shown in Fig. 2, the amplitude of endothelial Ca^2+^ responses and the extent of blood vessel relaxation increased with ACh concentration, and there was a positive relationship between the amplitude of endothelial Ca^2+^ responses and relaxations evoked by ACh. In SD and HFD groups, the line of best fit was linear and the gradient unity, suggesting a one-to-one relationship between population endothelial Ca^2+^ levels and vasodilator responses. Since the relationship between endothelial Ca^2+^ and relaxation was unchanged in obesity while relaxation was impaired, these findings suggest that endothelial dysfunction arises from an inability of the endothelium to generate a robust Ca^2+^ response in obese rats. Indeed, maximal ACh-evoked Ca^2+^ responses were lower in HFD (~60%) than in SD (~80%).

**Fig. 3:**
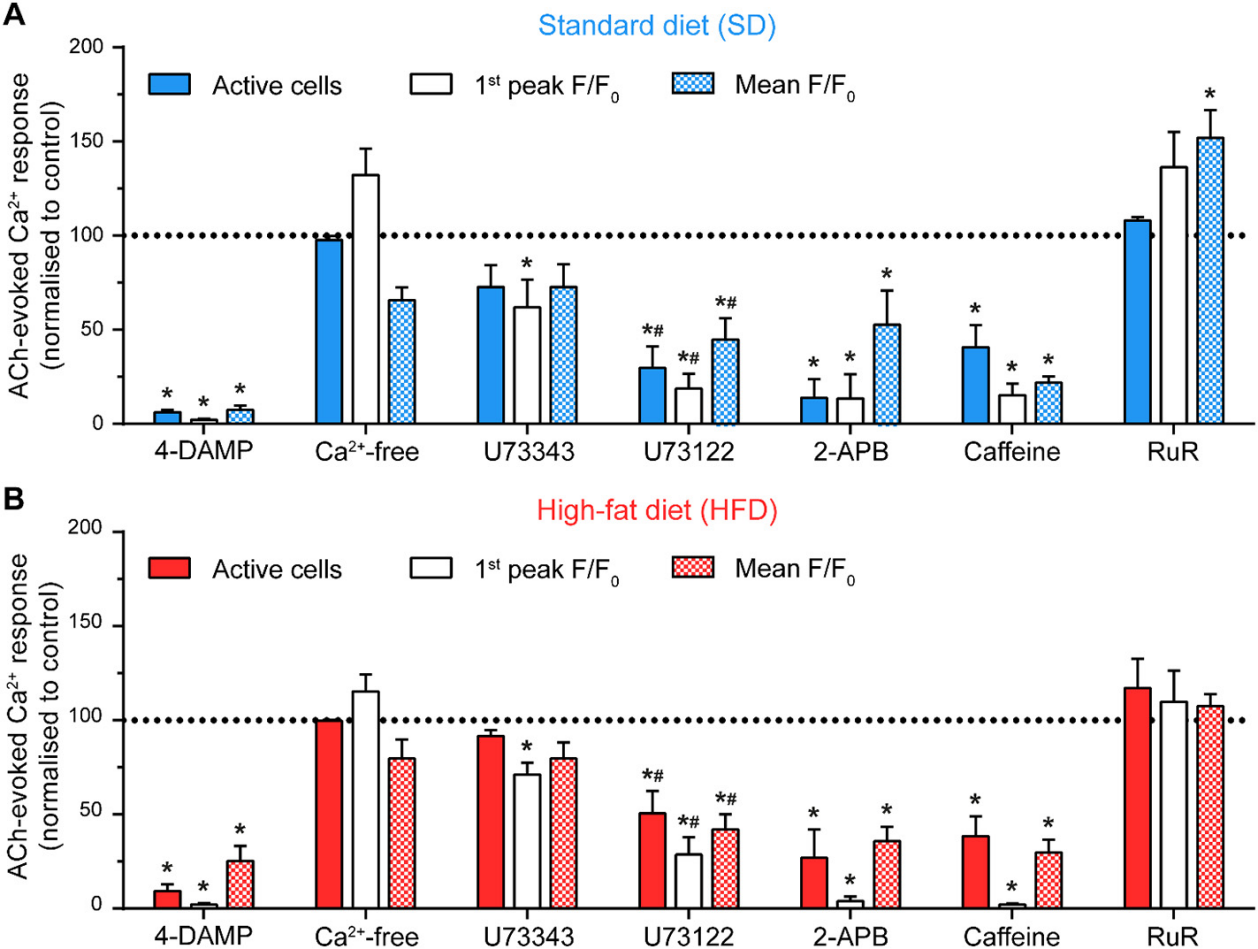
Acetylcholine activates endothelial Ca^2+^ signaling through the M_3_-PLC-IP_3_R pathway. Bar graph summarizing the effects of various pharmacological interventions on ACh (100 nM)-induced Ca^2+^ signaling in the endothelium of mesenteric arteries from rats fed either the standard diet (SD, A) or the high-fat diet (HFD, B). Ca^2+^-free PSS contained 1 mM EGTA. Concentrations of pharmacological inhibitors were: 4-DAMP (1 μM); U73343 (2 μM); U73122 (2 μM), 2-APB (100 μM); caffeine (10 mM); RuR (5 μM). All data are mean ± SEM expressed as a percentage of the control response in the same artery (response to ACh prior to pharmacological intervention). The effects of U73343 and U73122 were studied in the same vessels. * indicates p < .05 versus corresponding control, # indicates p < .05 versus corresponding U73343, using repeated measures two-way ANOVA (4-DAMP, Ca^2+^-free, 2-APB, caffeine, RuR) or repeated-measures two-way ANOVA with Tukey's multiple comparison test (U73343 and U73122). Data normalized to control for presentation. All statistical analyses used raw data. Data tabulated in Tables S5, S6.

To explore the mechanisms underlying the impaired Ca^2+^ response in obesity, we first determined the source of endothelial Ca^2+^ signals in our experimental model. To do this, the effects of several pharmacological interventions on various endothelial Ca^2+^ signaling parameters (number of activated cells, magnitude of the initial response, and magnitude of the response over 60 s) were examined (Fig. 3 and Tables S5, S6). In each experimental group (SD or HFD), ACh-evoked (100 nM) Ca^2+^ responses were inhibited significantly by the selective muscarinic M_3_ receptor antagonist, 4-DAMP (1 μM), consistent with previous studies demonstrating the role of the M_3_ receptor in the endothelial response to ACh [52]. In the absence of external Ca^2+^ (Ca^2+^-free PSS with 1 mM EGTA included in PSS), endothelial cells remained responsive to ACh, although the rise in cytoplasmic Ca^2+^ concentration was not sustained (Fig. S4). This result suggests that the initial response to ACh involved Ca^2+^ release from the internal store and that Ca^2+^ influx is required for the sustained phase. ACh-evoked responses were significantly reduced by the phospholipase C inhibitor, U73312 (2 μM), when compared to its inactive analogue U73343 (2 μM). Compared to control responses (no drug) and in contrast to U73312 (2 μM), U73343 (2 μM), did not reduce steady-state Ca^2+^ responses or the number of active cells although the first peak was reduced. Two IP_3_ receptor (IP_3_R) inhibitors, 2-aminoethoxydiphenyl borate (2-APB, 100 μM) and caffeine (10 mM; [[53], [54], [55]]), each inhibited ACh-evoked increases in Ca^2+^. The broad-spectrum transient receptor potential channel antagonist, ruthenium red (RuR, 5 μM), did not reduce ACh-evoked endothelial Ca^2+^ activity. These results demonstrate that ACh evokes a Ca^2+^ signalling cascade consisting of: 1) IP_3_-mediated Ca^2+^ release from the internal store; and 2) Ca^2+^ influx via store-operated Ca^2+^ entry [56,57]. As the magnitude of store-operated Ca^2+^ entry is dependent on that of the preceding Ca^2+^ release event, these results suggest that the impaired response in obesity arises from either reduced Ca^2+^ release from activated IP_3_ receptors or a reduction in the ability of IP_3_ to generate Ca^2+^ release.

**Fig. 4:**
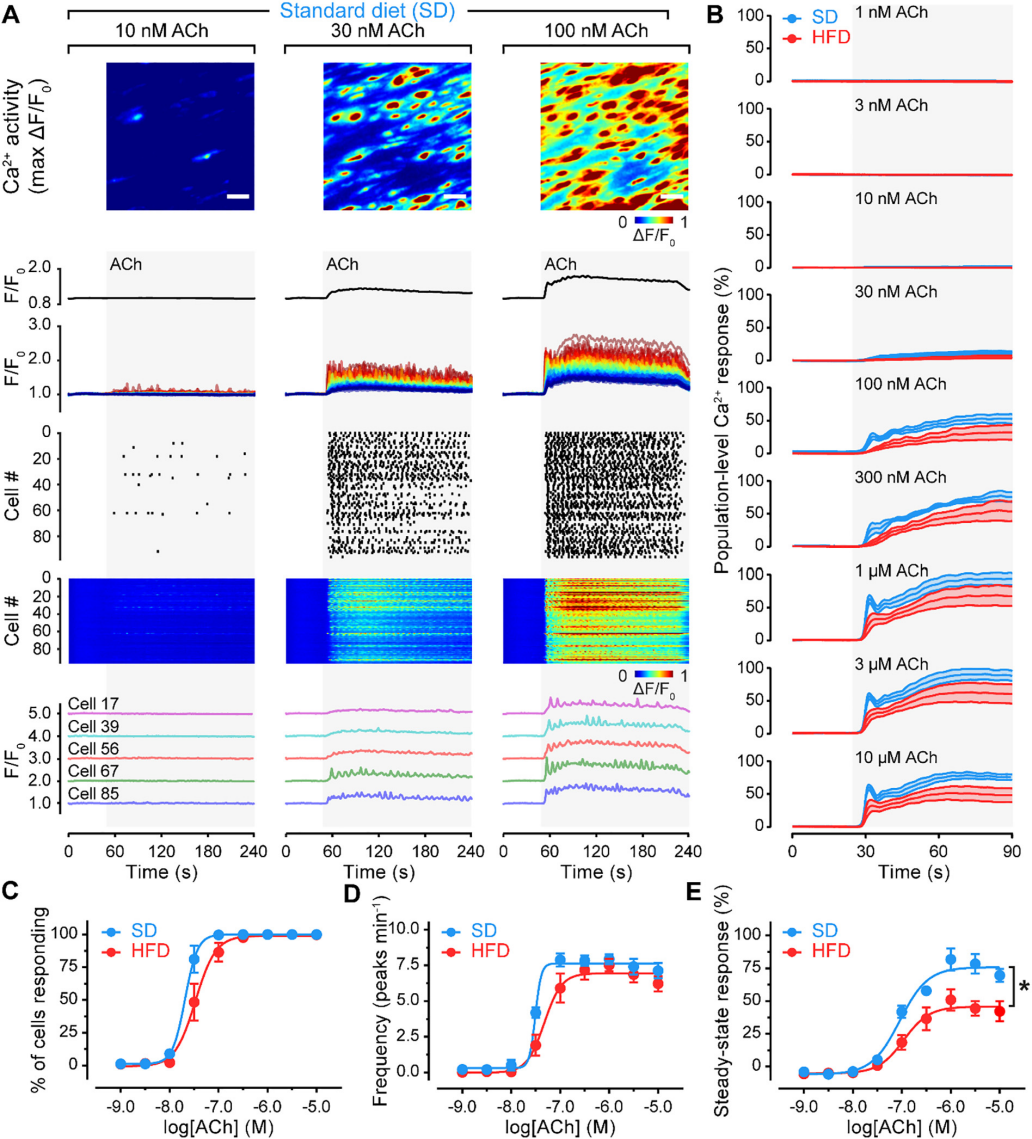
A high-fat diet impairs endothelial Ca^2+^ signaling. A) Concentration-dependence of ACh-evoked endothelial Ca^2+^ activity. Rows display (top to bottom): pseudocolored ΔF/F_0_ maximum intensity projections of a field of mesenteric endothelium stimulated with the indicated concentrations of ACh; average Ca^2+^ signal across the field of view; Ca^2+^ signals extracted from each cell; rastergrams; heatmaps; and example traces from five separate cells. B) Mean population-level responses to increasing concentrations of ACh in rats fed a standard diet (SD) or a high-fat diet (HFD). Data are mean ± S.E.M of n = 5 biological replicates, expressed as a percentage of the response to ionomycin. C–E) Summary of Ca^2+^ imaging data illustrating the concentration-dependence of the percentage of cells activated by ACh (C), the oscillation frequency (D), and the steady-state Ca^2+^ response (E) of SD and HFD rat endothelium. Data are mean ± SEM (n = 5 per group). * indicates significance (p < .05) by comparison of sigmoidal fit parameters (top of sigmoid) using ordinary two-way ANOVA with Sidak's multiple comparison test. HFD data shown in Fig. S3 and SD and HFD data tabulated in Table S7.

To investigate the mechanisms underlying alterations in IP_3_-mediated Ca^2+^ release in obesity, dynamic concentration-dependent activity from individual endothelial cells in intact blood vessels was examined. Spatial Ca^2+^ activity maps, generated from ΔF/F_0_ datasets show heterogeneity in the response of individual endothelial cells to ACh (Fig. 4). ACh-responsive cells were not uniformly distributed but scattered across the endothelium in clusters. Increasing ACh concentrations activated a progressively larger percentage of the endothelial cell population (Figs. 4A and S5A, top row). The concentration dependence of endothelial cell recruitment (% cells responding) was similar in the endothelium of SD and HFD groups (Fig. 4B and Table S7). These results show heterogeneity in the endothelial response in SD and HFD rats.

Temporal profiles of Ca^2+^ signals in individual cells (Figs. 4A and S5A, bottom rows) also show the response to ACh is heterogeneous across responding cells, and dependent on agonist concentration. To quantify ACh-evoked Ca^2+^ activity, we measured the steady-state Ca^2+^ level, the magnitude of Ca^2+^ oscillations, and the frequency of Ca^2+^ oscillations in each cell. The magnitude (steady-state response, amplitude of oscillations) of Ca^2+^ increases evoked by ACh were significantly impaired in the HFD when compared to the SD controls (Figs. 4D and S5C–D, and Table S7). However, there was no significant difference between the concentration-response relationship for Ca^2+^ signal oscillation frequency in HFD when compared to SD-fed rats (Fig. 4C and Table S7). Thus, in obesity although endothelial cells remain able to encode information in the frequency of Ca^2+^ signals, the amplitude of this activity is impaired.

One possible explanation for these results is that IP_3_ itself is less efficient in evoking Ca^2+^ release from internal stores in obesity. To examine this possibility, we bypassed PLC-dependent IP_3_ production using photolyzed caged-IP_3_ to directly activate IP_3_ receptors. Elevations in Ca^2+^ evoked by photolysis of caged-IP_3_ were similar in the SD and HFD groups (Fig. S6). This finding rules out the possibility that the reduced ACh-evoked endothelial Ca^2+^ activity in the HFD group arises from an impaired ability of IP_3_ to evoke Ca^2+^ release.

### 3.3 Abnormal endothelial Ca^2+^ responses reflect altered endothelial cell heterogeneity

The results presented so far suggest that obesity impairs vascular responses by reducing endothelial Ca^2+^ signaling, and that an inability of IP_3_ to evoke Ca^2+^ release does not explain the findings. Intracellular communication is critical in determining coordinated endothelial responses. Interactions among endothelial cells permit Ca^2+^ signals in discrete clusters of cells to coalesce into networked signaling patterns of activity that drive tissue-level responses [58,59]. Thus, we hypothesized that the endothelium may be unable to generate robust population-level Ca^2+^ responses in obesity because of either or both of: 1) alterations in the spatial distribution of endothelial sensitivity to ACh; and 2) altered communication between endothelial cells.

**Fig. 5:**
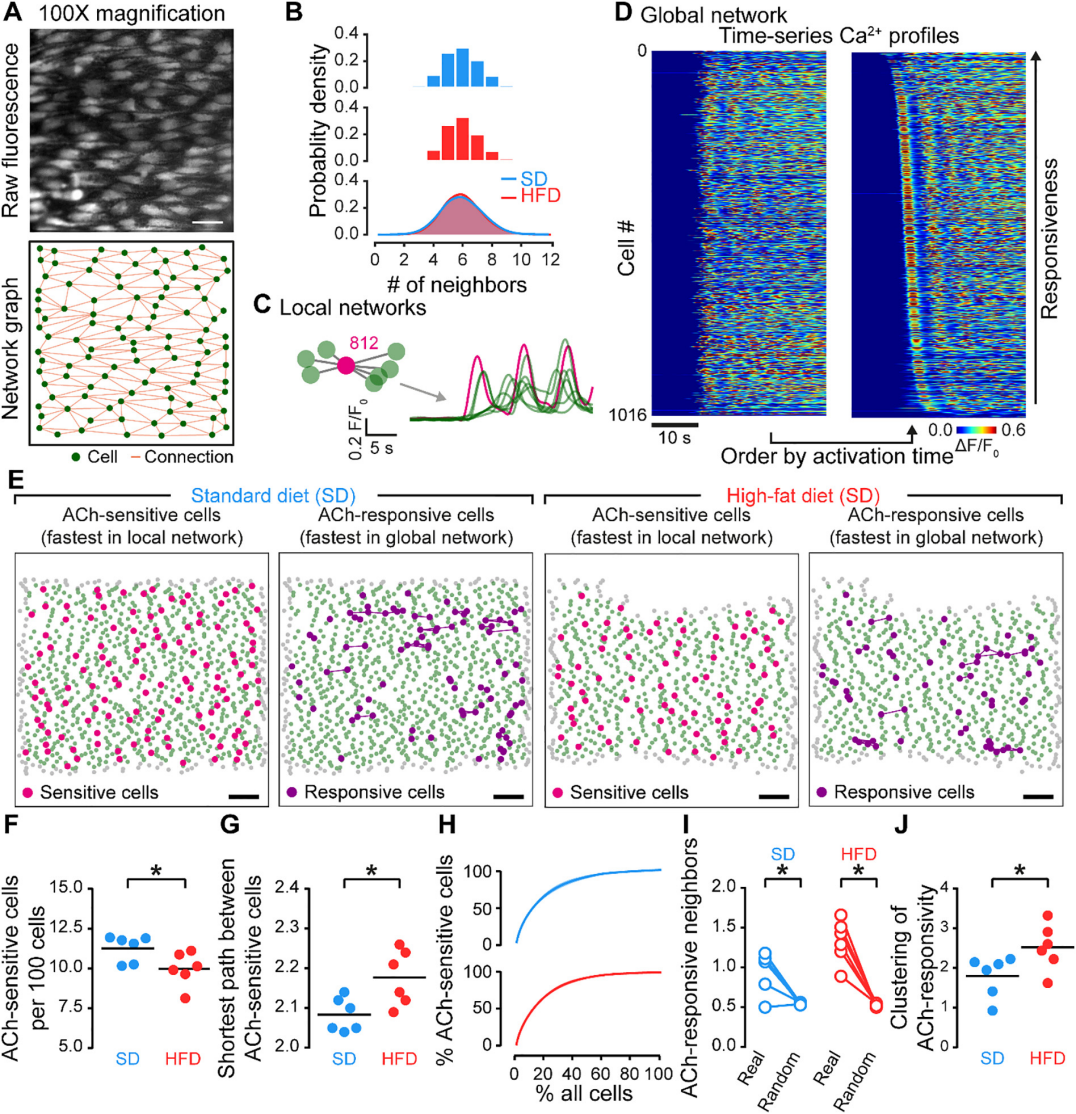
High-fat diet alters endothelial cell heterogeneity. A) High-resolution image of the endothelium and corresponding structural network. Connectivity computed using line segments regions-of-interest. Green circles indicate centroid locations, orange lines indicate connections between adjacent cells. B) Probability distribution of endothelial cell connectivity showing the number of neighbors each cell has. C–E) Endothelial heterogeneity was assessed using two methods. First, local cell networks were interrogated to reveal ACh-sensitive cells (those that respond to stimuli before any neighbor; C and E). Endothelial cells were also ranked by the time of their response to ACh to reveal the top 10% most ACh-responsive cells (D and E). Panel D shows a representative set of Ca^2+^ traces before (left) and after (right) temporal ranking. F–G) Summary data showing the effect of a high-fat diet on the density of ACh-sensitive cells (F) and the mean distance (number of cells) between ACh-sensitive cells (G). H) Graph showing that ACh-sensitive cells tend to be the first cells to respond. I–J) Summary data showing the clustering of ACh-responsive neighbors compared to an equivalent random model (I), and a comparison of clustering between SD and HFD groups (J). * indicates statistical significance (p < .05) using paired *t*-test (I) or unpaired *t*-test (F, G, J) with Welch's correction as appropriate. Scale bars = 20 μm (A) or 100 μm (E).

To test these possibilities, we examined the organization, functional relationships, and patterns of IP_3_-mediated Ca^2+^ activity occurring between neighboring endothelial cells (Fig. 5). In these experiments, large areas of endothelium (~0.8 mm^2^) were imaged and regions of interest were generated for each cell visualized (3667 cells, n = 6 for SD; 3051 cells, n = 6 for HFD). In both experimental groups, endothelial cells were arranged in a lattice network and each cell possessed an average of six immediate neighbors (Fig. 5A–C). This physical structure was unchanged in obesity. However, the structural connections among cells might give rise to altered functional connections in obesity. As a first step in examining the functional network in control and obesity, we identified cells that were unambiguously sensitive to ACh – i.e. those cells that responded to ACh before any immediate neighbor (ACh-sensitive cells; Fig. 5D&F). There was a smaller density of these ACh-sensitive cells in the HFD group compared to the SD group (Fig. 5G), and this generated an increased distance (path length) between ACh-sensitive cells (Fig. 5H). Thus, a significant change in endothelial responsiveness in obesity arises from a decreased density of agonist sensing cells.

To determine if endothelial cell clustering was altered in obesity, we next examined the distribution of agonist-responsive cells. In this analysis, we ordered cells by the speed at which they responded to ACh and identified the first 10% of cells to respond as ACh-responsive cells (Fig. 5E&F). Ca^2+^ responses in this fast-responding population of cells are likely due to direct agonist activation, rather than indirect activation arising from signals originating in neighboring cells and contained a large percentage of ACh-sensitive cells (Fig. 5F). In control and obesity, ACh-responsive cells had more ACh-responsive neighbors than would be expected if the endothelial cells were randomly distributed with respect to responsivity (Fig. 5J), i.e., ACh-responsive cells are clustered throughout the endothelial network [30,33]. Furthermore, compared to control, clustering of the ACh-responsive cell population was increased in obesity (Fig. 5J). This observation is significant as the increased clustering in HFD decreases the ability of responsive cells to engage with and recruit unresponsive cells across the network.

We next examined endothelial network communication by determining the functional connectivity of adjacent cells. In this analysis, a pairwise cross-correlation analysis was used to assess signal similarity between all neighboring endothelial cell pairs (i.e. quantifying the level of shared Ca^2+^ activity with time, Fig. S7). This type of analysis provides information on how cells encode information at the population level and how they interact, yielding insights into network communication mechanisms. In SD and HFD-fed animals, the mean pairwise correlation coefficients significantly exceeded chance levels (Fig. S7E). However, the extent of pairwise synchronicity was statistically similar in the two groups (Fig. S7F). This result indicates that the extent of communication between activated cells is unaltered in obesity. In support of this conclusion, Ca^2+^ wave propagation initiated by focal release of IP_3_ was similar in SD and HFD (Fig. S8). This finding also suggests that communication among cells is unaltered in obesity. Thus, whilst intercellular communication is unaltered, the density of ACh-sensitive cells is reduced, and the clustering of agonist-responding cells is increased in obesity.

### 3.4 Local Ca^2+^ signaling

An alternative route by which the endothelial network may be compromised is via changes in local signaling events. In many vascular diseases (e.g. hypertension [50]), dysfunctional endothelium-mediated responses arise from impaired signaling between endothelial and smooth muscle cells. Such signaling occurs via specialized myoendothelial projections (MEPs) that extend from endothelial cells, through holes in the internal elastic lamina (IEL), to smooth muscle cells. For example, IP_3_-evoked Ca^2+^ increases at MEPs may activate endothelial NO synthase and IK/SK channels [60,61], which each promote vascular relaxation. Because of this, we tested if the impaired vasodilation identified in HFD-fed rats might also reflect disruptions in endothelial-smooth muscle cell signaling. First, we determined the degree of myoendothelial connectivity by imaging the IEL (Fig. S9A–B). Holes in the IEL are a widely used measure of myoendothelial connectivity, although they do not definitively demonstrate the existence of MEPs. There was no clear change in IEL structure, as evidenced by similar IEL hole density, mean IEL hole size, and mean percentage area of IEL occupied by holes (IEL hole coverage; Fig. S9C–E and Table S8). We next examined the relationship between spontaneous Ca^2+^ activity and IEL holes (Fig. S10 and Table S8). In contrast to ACh-evoked Ca^2+^ activity, basal (unstimulated) Ca^2+^ events are mostly subcellular waves and the initiation site of individual events can be readily identified (see also [29,32]). Importantly, irrespective of diet, we found that endothelial Ca^2+^ events occurred closer to MEPs than would be expected than if they occurred randomly throughout the cytoplasm (Fig. S10C–D and Table S8). However, there was no difference in the extent of endothelial Ca^2+^-event-MEP coupling in SD- and HFD-fed rats (Fig. S10E and Table S8). Thus, it is unlikely that alterations in myoendothelial coupling contributes to the vascular dysfunction in obesity observed in the present study.

## 4 Discussion

Obesity, a disease characterized by excess body fat, is associated with type 2 diabetes, metabolic syndrome and cardiovascular diseases such as hypertension and stroke. Impaired endothelial function is a hallmark of obesity-related cardiovascular diseases [4], but mechanisms underlying the dysfunction are poorly understood. Here, we identify aberrant population-level activity in native endothelial cell networks of obese rats. Altered endothelial network circuits arose from compromised cell heterogeneity and increased clustering of sensory cells. The altered network circuits resulted in deficient encoding of vasoactive stimuli into population-level Ca^2+^ responses. Functionally, the network deficit manifested as impaired vasodilator responses. Abnormal vasodilator function occurred despite a lack of effect on intercellular Ca^2+^ signal propagation or pairwise synchrony between adjacent endothelial cells, suggesting a spatial network disruption rather than failure in network communication. Together, these findings: 1) highlight the role of population-level interactions in governing endothelial function, 2) offer functional insight into the endothelial cell heterogeneity seen in many genetic studies; and 3) provide support for a new cellular heterogeneity and organization hypothesis of endothelial dysfunction in cardiovascular disease.

Endothelial cells are interlaced with one another to form a regular mesh (hexagonal lattice) network in which each cell has ~6 adjacent neighbors. This structural architecture is unaltered by obesity. However, recent application of network theory to endothelial cell populations has started to uncover how rich functionality emerges from these structural connections and how network connectivity permits multiple physiological processes to be controlled simultaneously [[30], [31], [32], [33]]. Observing agonist-mediated endothelial Ca^2+^ activity in individual cells at the population level (hundreds to thousands of cells) reveals spatial modules, or clusters of sensory cells, that detect activators and recruit agonist-insensitive cells to drive network activity. Recruitment of agonist-insensitive cells occurs via the transmission and propagation of Ca^2+^ signals and increases the overall population of active cells contributing to the vascular response. Here, we show that the size of the sensory cell population is reduced in obesity and there is an increase in the clustering of agonist-responsive cells, i.e., in obesity there are fewer but larger modules. Increased clustering of sensory cells results in activated cells communicating to a greater extent with other agonist-activated cells. Since endothelial Ca^2+^ signals decay with transmission distance [[62], [63], [64]], the increased clustering limits recruitment of agonist-insensitive cells. Thus, changes in endothelial cell distribution disrupt collective endothelial cell behavior, reduce population-level Ca^2+^ responses and so impair endothelial vasodilator responses.

While widely acknowledged to exist (e.g. [34,65,[66], [67], [68], [69]]), the functional significance of endothelial cell heterogeneity is only beginning to emerge. In some tissues, heterogeneous cell populations organize into spatial domains with coherent gene expression [70], and the resulting multicellular groupings form local microcircuits, the building blocks of information processing. Clustering of endothelial cells also permits the distribution of activities across space, i.e., different functional units can perform different processes simultaneously [[30], [31], [32]]. Such parallel processing requires cells that are responding to one stimulus to be resilient to interference from neighboring cells and this is facilitated by the organization of cells into clusters. Clustering will also increase the concentrations of diffusible messengers (e.g. NO), so increasing their effective range, by overwhelming local breakdown mechanisms [31]. While we provide functional data supporting a role for cell clustering in endothelium-dependent vasodilation, a number of studies suggest that the impact of spatial heterogeneity extend beyond the control of vascular tone. For example, leaky sites in inflamed blood vessels are highly localized, suggesting that endothelial permeability may be regulated by distinct cell populations [71]. Similarly, imaging studies that have identified distinct subpopulations of aortic endothelial cells with self-renewal capacity suggest a role for local heterogeneity in tissue repair [72,73]. Further studies will be required to definitively establish a functional role of endothelial cell heterogeneity in each of these processes.

Endothelial cells in different vascular beds are also heterogeneous and endowed with specific functions suited to the organs they vascularize [74]. There is also heterogeneity in the mechanisms that give rise to specific functions. For example, the relative contributions of NO and endothelial-derived hyperpolarization to vasodilator responses differ in systemic and pulmonary circulations [75]. The contributions of these Ca^2+^-dependent dilator mechanisms differ also within specific vascular beds and depend on blood vessel calibre [[76], [77], [78], [79]]. In this regard, localized Ca^2+^ signaling at MEPs appears important for the relaxation of smooth muscle via endothelium-derived hyperpolarization in small mesenteric arteries [61], but not in larger arteries. In the present study, there was no differences in the distribution of IEL holes or the extent of endothelial Ca^2+^-event-IEL hole coupling in SD- and HFD-fed rats. These findings suggest that alteration in myoendothelial coupling is unlikely to contribute to the vascular dysfunction in obesity observed in the present study. Notwithstanding, the activation of the vasodilator pathway NO or endothelial-derived hyperpolarization may depend on the nature [80] or extent [81] of the vasoconstrictor stimulus (e.g. adrenergic receptor stimulation vs myogenic tone) or the specific Ca^2+^ signaling modalities engaged by the vasodilator stimulus. Here, we demonstrate a role for endothelial cell heterogeneity in the agonist-evoked, NO-mediated relaxation of preconstricted first-order mesenteric arteries. Future studies will determine how cell heterogeneity plays a role in other endothelial vasodilator pathways.

In the present study, despite the unambiguous existence of local heterogeneity in cell organization and Ca^2+^ responses, the relationship between population-level endothelial Ca^2+^ signaling and vasodilator function is linear and injective. Precisely how endothelial microcircuits interact and coordinate to control vascular reactivity via tissue-level endothelial Ca^2+^ signaling is an important question, particularly as the mapping of endothelial Ca^2+^ levels to vascular relaxation is unaltered in obesity. Cooperativity among cells is essential for generating coherent population-level responses and necessitates communication among endothelial cells. Such communication is achieved in endothelial cell networks via the transmission of IP_3_ or Ca^2+^ or both, through intracellular gap junctions that connect neighboring cells [58,82]. Two findings indicate that, in obesity, endothelial cells remain connected and retain the ability to communicate with each other. First, the extent of pairwise similarity between Ca^2+^ signals of neighboring cells was similar in control and obesity, suggesting comparable functional connectivity. Second, evoked Ca^2+^ wave propagation was similar in control and obesity. Consistent with a cell heterogeneity hypothesis of endothelial dysfunction, these findings suggest that the mechanisms underlying endothelial cell cooperativity are largely unaltered in obesity.

Our finding that the density of agonist-sensitive endothelial cells is decreased, while clustering of agonist-responsive cells is increased, demonstrates that altered endothelial cell heterogeneity and network organization are responsible for deficient vascular function in obesity. Recent single-cell RNA sequencing studies of whole aorta have also identified distinct endothelial cell subpopulations [83]. These subpopulations have gene expression patterns that are suggestive of functional specialization (e.g. angiogenesis, inflammatory responses, vasomotor control, extracellular matrix production) and are modulated by diet and blood pressure [[83], [84], [85]]. We have not addressed the mechanisms responsible for promoting altered endothelial cell clustering in the present study. However, our previous studies provide evidence that clustering of agonist responsive cells occurs via variations in receptor expression [30,33]. Heterogeneity in the expression patterns or subcellular distributions of the Ca^2+^ signaling components, such as the various IP_3_R isoforms [86], or components of store operated Ca^2+^ entry such as STIM and ORAI [56,[87], [88], [89]] may each also contribute. Increased oxidative stress underlies the vascular changes in several cardiovascular diseases. Interestingly, the inhibitory effect of oxidative stress induced by hydrogen peroxide, on spontaneous and agonist-evoked Ca^2+^ signals, is also heterogeneous across the endothelium [90]. This observation raises the possibility that increased oxidative stress may reorganize Ca^2+^ signaling patterns in obese animals [16,17].

Molecular and functional diversity in endothelial cells may arise from local microenvironmental factors. In support, transplanted endothelial cells gain structural phenotypes and gene expression patterns associated with new host tissue [67,91] and endothelial cells with distinct molecular signatures regress towards a common phenotype upon in vitro expansion [39,92]. In obesity, differences in the local environment may also arise from variation in lipid accumulation among endothelial cells [93]. Heterogeneity has also been reported to occur from the specific origins of endothelial cells rather than the local environment. Two distinct developmental origins give rise to endothelial cells in mature blood vessels [94]. Heritable factors from these different origins may explain why proliferative endothelial cells exist alongside cells with a lower proliferative potential [72,73], and give rise to clusters of endothelial cells with similar properties via clonal expansion [37,38]. Interactions between heritable factors and local environmental conditions may also explain endothelium dysfunction in obesity. Clarifying how functional endothelial cell heterogeneity emerges and is impacted by disease states such as obesity is a major area for future work.

In conclusion, the present results show that the endothelium is a collection of exquisitely organized cells, and that this organization governs endothelial function. Impaired vascular function in obesity arises from alterations in endothelial cell heterogeneity, specifically the altered distribution of sensory cells, and results in deficient network-level function (vasodilation). As the mechanisms disrupting endothelial network function are not yet clear, significant work remains to address a heterogeneity model of vasomotor dysfunction. Future studies will investigate drivers of altered endothelial heterogeneity and network dysfunction in obesity and its related vascular diseases. As collective cell behavior also controls the regeneration of injured endothelium [73], the consequences of network dysfunction are likely to extend far beyond the control of blood vessel diameter. Understanding the full functional significance of endothelial cell network organization will provide a deeper understanding of the pathophysiology of vascular disease.

## Author contributions

JGM & CW developed the concept. NMNA & SD developed the animal model. CW, XZ, MM, MDL, CB & HRH performed the experiments, and analyzed the data. CW, XZ, MDL, CB, HRH, & JGM interpreted the data. CW & JGM drafted the manuscript. All authors revised and edited the manuscript and approved the final version of the manuscript.

## Declaration of competing interest

None.

## Appendix A Supplementary data

Supplementary material Image 1
